# Latent tuberculosis infection and associated risk indicators in pastoral communities in southern Ethiopia: a community based cross-sectional study

**DOI:** 10.1186/s12889-018-5149-7

**Published:** 2018-02-17

**Authors:** Takele Teklu, Mengistu Legesse, Girmay Medhin, Aboma Zewude, Mahlet Chanyalew, Martha Zewdie, Biniam Wondale, Milkessa Haile-Mariam, Rembert Pieper, Gobena Ameni

**Affiliations:** 1 Aklilu Lemma Institute of Pathobiology (ALIPB), Addis Ababa University, P O Box 1176, Addis Ababa, Ethiopia; 2 Department of Immunology and Molecular Biology, College on Medicine and Health Sciences, University of Gondar, Gondar, Ethiopia; 3 Armauer Hansen Research Institute, AHRI, PO Box 1005, Addis Ababa, Ethiopia; 4 Department of Biology, College of Natural Sciences, Arbaminch University, Arbaminch, Ethiopia; 5 Department of Veterinary Laboratory, College of Agriculture, Ambo University, Guder, Ethiopia; 6 J. Craig Venter Institute, Rockville, MD USA

**Keywords:** LTBI, IGRA, Prevalence, South Omo, Ethiopia

## Abstract

**Background:**

Research pertaining to the community-based prevalence of latent tuberculosis infection (LTBI) is important to understand the magnitude of this infection. This study was conducted to estimate LTBI prevalence and to identify associated risk factors in the Omo Zone of Southern Ethiopia.

**Methods:**

A community-based cross-sectional study was conducted in six South Omo districts from May 2015 to February 2016. The sample size was allocated to the study districts proportional to their population sizes. Participants were selected using a multi-stage sampling approach. A total of 497 adult pastoralists were recruited. Blood samples were collected from the study participants and screened for LTBI using a U.S. Food and Drug Administration approved interferon-gamma release assay (IGRA). Logistic regression was used to model the likelihood of LTBI occurrence and to identify risk factors associated with LTBI.

**Results:**

The prevalence of LTBI was 50.5% (95% CI: 46%, 55%) with no significant gender difference (49.8% among males versus 51.2% among females; Chi-square (χ^2^) = 0.10; *P* = 0.41) and marginally non-significant increasing trends with age (44.6% among those below 24 years and 59.7% in the age range of 45-64 years; χ^2^ = 6.91; *P* = 0.075). Being residence of the Dasanech District (adjusted odds ratio, AOR = 2.62, 95% CI: 1.30, 5.28; *P* = 0.007) and having a habit of eating raw meat (AOR = 2.89, 95% CI: 1.09, 7.66; *P* = 0.033**)** were significantly associated with an increased odds of being positive for LTBI. A large family size (size of 5 to 10) has significant protective effect against associated a reduced odds of being positive for LTBI compared to a family size of below 5 (AOR = 0.65, 95% CI: 0.42, 0.99; *P* = 0.045).

**Conclusions:**

A high prevalence of LTBI in the South Omo Zone raises the concern that elimination of TB in the pastoral communities of the region might be difficult. Screening for and testing individuals infected with TB, independent of symptoms, may be an effective way to minimize the risk of disease spread.

## Background

While the directly observed treatment short-course (DOTS) strategy has achieved remarkable progress in tuberculosis (TB) control in many parts of the world [1], this disease continues to be a major concern to public health in the twenty-first century [2]. TB was a leading cause of morbidity and mortality from a single infectious agent in 2015 with 10.4 million new cases and 1.7 million deaths (0.4 million of which pertained to (human immune-deficiency virus (HIV) coinfection) [1]. This makes TB the ninth leading cause of death ranking above human immunodeficiency virus infection and acquired immune deficiency syndrome (HIV/AIDS) in the world [1]. Drug-resistant (MDR)-TB is a wide spread problem. Six hundred thousand new rifampicin resistant cases, 490,000 of which were multidrug resistance cases, were reported in 2016 alone [1].

Latent TB infection (LTBI) has been defined as a state featuring persistent immune responses to *Mycobacterium tuberculosis (Mtb)* antigens without evidence of the manifestation of clinical symptoms [3]. It is estimated that approximately two to three billion people living in high TB burden countries are infected with *Mtb* complex (MTBC) bacteria [1]. Of those, approximately 1.3 million will develop active TB during their lifetime [4]. Most of these patients develop active TB within the first 5 years unless they are diagnosed and treated with antibiotic drugs [4]. Recent investigations suggest that LTBI is a more complex phenomenon. The risk of progression to active TB appears to be high in some same-household groups. It was also reported that granulomas have different metabolic activity states in the same patient [5, 6]. Clinical tests to diagnose LTBI are of paramount importance to assess the risk of TB reactivation. World health organization (WHO’s) post-2015 global TB strategy framework adapted from the “End TB Strategy” states that systematic testing and treatment of LTBI in at-risk populations is a critical component in the elimination of TB [3].

African countries host approximately 60% of the world pastoralists. In the Horn of Africa, these groups are marginalized, impoverished and have little access to medical care [7]. Previous studies have revealed a high prevalence of LTBI in high-risk groups such as health care workers, prisoners and gold miners [8–11] but only a few pastoral community-based studies [12, 13] have been conducted. Investigating the prevalence of LTBI in such populations is important to evaluate the disease burden and determine if LTBI treatment policies need to be adjusted. LTBI can be diagnosed using a tuberculin test and or an interferon gamma release assay (IGRA) [14–16]. Although the IGRA is not a gold standard test for LTBI [15], the studies recommended that version of the IGRA are suitable to estimate the extent of LTBI in a human subject under study [17, 18]. The present study was undertaken to assess the prevalence of LTBI using IGRAs and to identify associated risk factors in pastoral communities of the South Omo Zone of southern Ethiopia.

## Methods

### Study design and study area

**Fig. 1:**
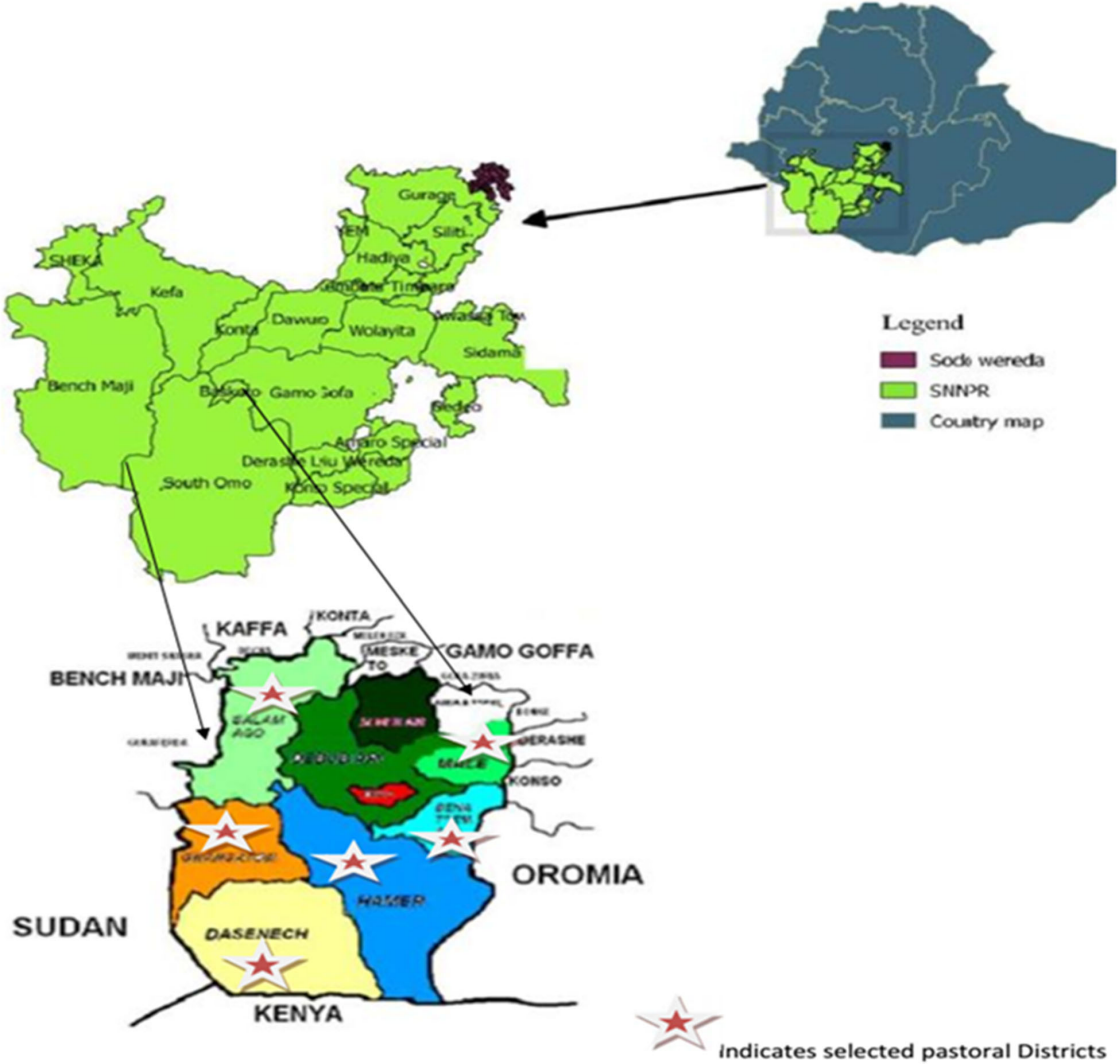
Spatial Distribution of study sites in South Omo pastoral communities, southern Ethiopia

A community-based cross-sectional study was carried out in South Omo, a Zone in southern Ethiopia, from May 2015 to February 2016 to estimate the prevalence of LTBI among pastoral communities. This Zone shares borders with Kenya, South Sudan, Gamo Gofa Zone and Oromia Regional State (Fig. 1) [19]. It has a total area of 21,056 km^2^ with 573,435 population. South Omo has eight districts, the majority of the population live in Hamer District (25%) and pastoralists live in six of the eight districts. The inhabitants of the Zone are classified into 16 indigenous ethnic groups with the Dassanech as the most dominant group [20].

The available health facilities include one General Hospital, 32 health centers, and 225 health posts. Of these health facilities, the General Hospital and 23 health centers provide acid fast bacilli microscopy examinations and TB treatment, while the other nine health centers provide only TB treatment. In 2016, 58 additional health care posts started a DOTS programme. The TB detection rate was 64% in 2016 but TB was still not included in the list of top ten diseases in the Zone [21].

### Sample size determination and data collection

The sample size for the study was estimated by assuming a 63.7% prevalence of LTBI [12], 95% confidence interval, 5% margin of error and 1.26 design effect [22]. The non-respondent rate was estimated to be 10% resulting in a minimum required sample size of 497.

Participants were selected using a multi-stage sampling approach. In order to increase both the efficiency of sampling and the precision, the total sample size was distributed to the districts proportional to their population sizes. Accordingly, 128 participants were from Hamer, 116 were from Benatsemay, 105 were from Dasanech, 62 were from Selamago, 42 were from Malee and 44 were from Ngangatom. Several kebeles (sub-districts) corresponding to the number of study participants allocated to each district were randomly sampled using a computer-based random number generator. The list of participants was taken from health facility of each district. Study participants from each household were enrolled from those individuals who were at home during the data collection.

Individuals were eligible for participation if they did not have signs and symptoms of TB, were at least 18 years of age and not pregnant in case of females. Volunteers were informed of the study’s purpose, consented, provided three ml blood and underwent clinical and physical examinations including inspection for scars associated with bacilli Calmette-Guerin (BCG) vaccination and anthropometric measures to check nutritional status. Each study participant was interviewed face-to-face to gather information on her/his medical history including contact with TB patients, other acute or chronic disease and drug treatments for them, the number of family members, smoking, Khat chewing and alcohol consumption habits, prior imprisonment and hospitalizations, raw milk and meat consumption and sharing beverages. HIV test results were available for some individuals from health facility records. Socio-demographic characteristics of study participants were included in the questionnaire.

### QuantiFERON-TB Gold In-Tube assay

The QuantiFERON-TB Gold In-Tube test (GFT-GIT) was performed according to the manufacturer’s instructions (QuantiFERON-TB Gold In-Tube, Cellestis Ldt., Carnegie, Australia). In brief, 3 ml of a venous blood was collected from each study participant and1ml was put into each of three tubes labelled as ‘nil’, ‘TB-specific antigens’, and ‘mitogen’. The blood sample was transported to Jinka Regional Laboratory within 8 h of collection. Prior to incubation, all tubes were maintained at room temperature and re-mixed by inverting 10 times, then incubated for 24 h at 37 °C. The cultures were centrifuged for 15 min at 3000 relative centrifugal force (g) after which the plasma was harvested and stored at − 80 °C. Frozen samples were thawed and IFN-γ release was measured using the QuantiFERON-TB enzyme linked immunosorbent assay (ELISA) protocol. Sample absorbance was read at a lambda maximum of 450 nm with a reference wavelength of 620 nm using appropriate settings in a 96-well plate spectrophotometer. Results were interpreted as positive, negative or indeterminate with a cut-off value of interferon gamma (IFN-γ) > 0.35 international unite per milliliter (IU/ml) using QuantiFERON ®-TB Gold analysis software version 2.62 (Cellestis, Carnegie, Australia, http://www.cellestis.com).

### Data analysis

Data were entered into EpiData version 3.1 and statistical package for the social sciences (SPSS) version 20.1 software tools. The primary outcome was LTBI status recorded as present or not present, defining a concentration of IFN-γ ≥ 0.35 IU/ml as presence of LTBI. The overall prevalence of LTBI was estimated by dividing the number of participants with the concentration of IFN-γ ≥ 0.35 IU/ml by the total number of study participants who had undergone the QFT-GIT test. Frequencies and percentages were used to summarize characteristics of study participants. The Pearson Chi-square (χ^2^) was used to test the difference in proportions of LTBI across the categorical variables. Bivariate logistic regression analysis was performed to obtain crude odds ratio (OR) with corresponding 95% confidence intervals (95% CI). Multiple logistic regression analysis was performed to assess simultaneously the association between multiple risk factors and the log odds of being positive for LTBI. From this model, adjusted odds ratios (AOR) and 95% CI were obtained. The linear regression method was used to assess the effects of individual-level confounders like gender, age, BMI, health status, any drug treatments during blood sample collection and vaccination with BCG on IFN-γ response.

### Ethics considerations

Ethical approval for the study was obtained from Addis Ababa University, Aklilu Lemma Institute of Pathobiology Research and Ethics Committee as well as from the National Research Ethic Review Committee of Ethiopia (Ref No:3.10/785/07). Written consent was obtained from each study participant after a clear explanation of the study objectives. Blood sample collection was undertaken under aseptic conditions by licensed medical laboratory professionals. Volunteers with any sign and symptom of active TB or any other diseases during data collection time were transported at the project’s expense to nearby health facilities to undertake a complete examination. Individuals who had LTBI were advised to consult nearby health facilities regarding the development of symptoms of active TB.

## Results

### Socio-demographic characteristics of the study participants

As shown in Tables 1, 497 study participants (the age ranged from 18 to 80, mean age 37.2) were enrolled 50.5% of whom were males and 50.4% of whom were within the age range of 25 to 44 years. The majority (76.7%) were married and 84.5% were not formally educated.

**Table 1:** Socio-demographic characteristics of the study participants (*N* = 497)

Characteristics		Number (%)
Gender:	Male	251 (50.5)
Age (in years) (*N* = 494)	≤ 24	84 (17.0)
	25-44	249 (50.4)
	45-64	150 (30.4)
	65+	11 (2.2)
Religion:	Christianity	151 (30.4)
	Cultural belief	346 (69.6)
Marital status	Married	381 (76.7)
	Unmarried	56 (11.3)
	Others	60 (12.1)
Educational status	Literate study participants	77 (15.5)
Occupation	Pastoral	412 (82.9)
Residents	Rural	464 (93.4)
District	Benatsemay	116 (23.3)
	Hamer	128 (25.8)
	Dasanech	105 (21.1)
	Ngangatom	44 (8.9)
	Salamago	62 (12.5)
	Malee	42 (8.5)
Health status	Sick	129 (26.0)
Currently using antibiotic for any illness	Yes	18 (3.6)
BMI (*N* = 478)	< 18.5	153 (32.0)
	18.5-24.99	308 (64.4)
	≥ 25	17 (3.6)
TB contact history	Yes	165 (33.2)
	No	322 (64.8)
	Unknown	10 (2.0)
Number of family members	< 5	302 (60.8)
	5-10	165 (33.2)
	> 10	30 (6.0)
Tobacco smoker (*N* = 486)	Yes	265 (54.5)
Alcohol user	Yes	354 (71.2)
Hospitalization	Yes	65 (13.1)
Imprison	Yes	33 (6.6)
BCG Scar	Yes	416 (83.7)
Raw milk consumption	No	39 (7.8)
	Yes sometimes	77 (15.5)
	Yes always	381 (76.7)
Raw meat consumption	No	145 (29.2)
	Yes sometimes	313 (63.0)
	Yes always	39 (7.8)
Sharing of drinking materials with TB patients	Yes	38 (8.1)

Note *BMI* Body Mass Index, *BCG* Bacille Calmette Guerin

### IFN-γ response to *Mtb* specific antigens

The concentration of IFN-γ (TB antigen minus the nil) was more than 10 IU/ml in 194 individuals, in the range of 5-10 IU/ml in 18 individuals, in the range of 1-5 IU/ml in 18 individuals, in the rage of 0.35-1 IU/ml in 21 individuals and < 0.35 IU/ml in 230 individuals. The results were indeterminate in 16 (3.2%) of the cases. None of the individual-level variables investigated during the analysis had significant effect on the IFN-γ response to *Mtb* specific antigens before and after adjusting for the remaining variables (*P* > 0.05) (Table 2).

**Table 2:** Evaluating the effects of selected individual-level associated factors on the level of IFN-γ

Characteristic	Crude mean difference (95%CI)	Adjusted mean difference (95%CI)	*P*-Value
Gender	0.408 (−0.325-1.140)	0.300 (−0.460-1.061)	0.438
Age	0.010 (−0.018-0.038)	0.010 (−0.019-0.040)	0.490
BMI	−0.259 (− 0.972-0.454)	−0.196 (− 0.926-0.534)	0.598
Health status	0.516 (−0.321-1.353)	0.517 (− 0.358-1.393)	0.246
Current antibiotic use	0.765 (−1.188-2.714)	0.865 (−1.176-2.907)	0.405
BCG	0.429 (−0.559-1.418)	0.275 (−0.758-1.309)	0.601

Reference categories were male for gender, sick for health status, current antibiotic users for current antibiotic use and vaccinated for BCG vacination status

Note *BMI* Body Mass Index, *BCG* Bacille Calmette Guerin

### Prevalence of LTBI

The prevalence of LTBI was 50.5% (95% CI: 46%, 55%) with no significant gender difference (49.8% among males and 51.2% among females; χ^2^ = 0.10; *P* = 0.41). Although the prevalence of LTBI increased from 45.2% among those below 24 years to 54.5% in the age range of 45-64 years, this increase was not statistically significant (χ^2^ = 6.91; *P* = 0.075). The prevalence was higher in the Dasanech District than in the Benatsemay District (64.8% vs 41.4%; χ^2^ = 15.17; *P* = 0.010) and among those who reported eating raw meat frequently versus those who did not (66.7% vs 52.4%; χ^2^ = 8.1; *P* = 0.042) (Table 3). Two hundred and six individuals reported previous HIV testing; three of them were HIV positive. Two of the three HIV positive subjects had a positive LTBI result.

**Table 3:** Risk indicators for LTBI in South Omo pastoral communities

Variables		With LTBI	Without LTBI	COR (95%CI)	AOR (95%CI)	P-value
		No (%)	No (%)			
Gender	Male	125 (49.8)	126 (50.2)	Ref		
	Female	126 (51.2)	120 (48.8)	1.06 (0.75-1.51)	1.23 (0.80-1.90)	0.350
Age (in year)	≤ 24	38 (45.2)	46 (54.8)	Ref		
	25-44	117 (47.0)	132 (53.0)	1.07 (0.65-1.76)	0.96 (0.50-1.84)	0.904
	45-64	89 (59.3)	61 (40.7)	**1.77 (1.0-3.03)**	1.40 (0.67-2.94)	0.376
	65+	6 (54.5)	5 (45.6)	1.45 (0.41-5.13)	1.43 (0.32-6.50)	0.640
Religion	Christian	87 (57.6)	64 (42.4)	Ref		
	Others	164 (47.4)	182 (52.6)	**0.66 (0.45-0.96)**	0.67 (0.39-1.23)	0.211
Marital status	Married	195 (51.2)	186 (48.8)	Ref		
	Unmarried	23 (41.1)	33 (58.9)	0.86 (0.50-1.48)	0.63 (0.29-1.38)	0.249
	Others	33 (55.0)	27 (45.0)	0.57 (0.27-1.19)	0.99 (0.50-1.95)	0.976
Education status	Illiterate	211 (50.2)	209 (49.8)	Ref		
	Literate	40 (51.9)	37 (48.1)	1.07 (0.66-1.74)	1.42 (0.71-2.85)	0.326
Occupation	Pastoral	208 (50.5)	204 (49.5)	Ref		
	Other	43 (50.6)	42 (49.4)	1.00 (0.63-1.60)	0.77 (0.38-1.55)	0.463
District	Benatsemay	53 (41.4)	75 (58.6)	Ref		
	Hamer	52 (44.8)	64 (55.2)	1.15 (0.69-1.91)	1.15 (0.59-2.22)	0.682
	Dasanech	68 (64.8)	37 (35.2)	**2.35 (1.36-4.05)**	**2.62 (1.30-5.28)**	**0.007**
	Ngangatom	24 (54.5)	20 (45.5)	1.70 (0.85-3.39)	1.30 (0.43-3.99)	0.641
	Selamago	34 (54.8)	28 (45.2)	1.72 (0.93-3.12)	1.77 (0.86-3.65)	0.123
	Malee	20 (47.6)	22 (52.4)	1.29 (0.64-2.59)	1.49 (0.65-3.59)	0.344
Health status	Normal	177 (48.1)	191 (51.9)	Ref		
	Sick	74 (57.4)	55 (42.6)	1.45 (1.97-2.17)	1.24 (0.76-2.02)	0.393
BMI	< 18.5	88 (57.5)	65 (42.5)	Ref		
	18.5-24.99	147 (47.7)	161 (52.3)	0.67 (0.46-1.00)	0.75 (0.49-1.19)	0.221
	> 25	7 (41.2)	10 (58.8)	0.52 (0.19-1.43)	0.54 (0.17-1.76)	0.307
Contact history	Yes	90 (54.5)	75 (45.5)	Ref		
	No	154 (47.8)	168 (52.2)	0.76 (0.52-1.11)	0.74 (0.47-1.17)	0.201
	Unknown	7	3	1.94 (0.49-7.78)	1.99 (0.44-9.02)	0.374
Number of family	< 5	165 (54.6)	137 (45.4)	Ref		
	5-10	72 (43.6)	93 (56.4)	**0.64 (0.44-0.94)**	**0.65 (0.42-0.99)**	**0.045**
	> 10	14 (46.7)	16 (53.3)	0.73 (0.34-1.54)	0.73 (0.31-1.73)	0.479
Tobacco smoking	Yes	138 (52.1)	127 (47.9)	Ref		
	No	108 (48.9)	113 (51.1)	0.89 (0.62-1.27)	1.08 (0.69-1.70)	0.736
Alcoholism	Yes	184 (52.0)	170 (48.0)	Ref		
	No	67 (46.9)	76 (53.1)	0.82 (0.55-1.20)	0.70 (0.43-1.14)	0.150
Hospitalization	Yes	31 (47.7)	34 (52.3)	Ref		
	No	220 (50.9)	212 (49.1)	1.12 (0.68-1.92)	1.05 (0.57-1.94)	0.872
Imprison	Yes	15 (45.5)	18 (54.5)	Ref		
	No	236 (50.9)	228 (49.1)	1.24 (0.61-2.53)	1.37 (0.59-3.20)	0.872
Raw milk consumption	No	20 (51.3)	19 (48.7)	Ref		
	Yes sometimes	46 (59.7)	31 (40.3)	1.41 (0.64-3.06)	1.24 (0.49-3.13)	0.649
	Yes always	185 (48.6)	196 (51.4)	0.90 (0.46-1.73)	0.63 (0.28-1.40)	0.255
Raw meat consumption	No	76 (52.4)	69 (47.6)	Ref		
	Yes sometimes	149 (47.6)	164 (52.4)	0.83 (0.57-1.22)	1.02 (0.56-1.88)	0.946
	Yes always	26 (66.7)	13 (33.3)	1.82 (0.87-3.81)	**2.89 (1.09-7.66)**	**0.033**
BCG Scar	No	38 (46.9)	43 (53.1)	Ref		
	Yes	213 (51.2)	203 (48.8)	1.19 (0.74-2.91)	0.95 (0.54-1.67)	0.869
Sharing of drink materials with TB Patients	No	226 (52.8)	203 (47.2)	Ref		
	Yes	24 (63.2)	14 (36.8)	1.53 (0.77-3.04)	1.13 (0.71-2.99)	0.847

Note *No* number, *BMI* Body Mass Index, *BCG* Bacille Calmette Guerin

Boldfaces are the variable that showed statistically significant either in crude and/or adjusted odds ratios

### Risk factors for LTBI in South Omo pastoral communities

The results from a logistic regression taking log-odds of LTBI positive as an outcome variable are summarized in Table 3. The odds of having LTBI were higher among individuals living in the Dasanech District (OR = 2.35; 95% CI: 1.36, 4.05; AOR = 2.62, 95% CI: 1.30, 5.28; *P* = 0.007) compared to those living in the Benatsemay District and among individuals who frequently ate raw meat (OR = 1.8 95% CI: 0.87, 3.81; AOR = 2.89, 95% CI: 1.09, 7.66; *P* = 0.033) compared to those who did not. The odds of LTBI positivity was smaller among individuals with a small family size (OR = 0.64 95% CI: 0.44, 0.94; AOR = 0.65, 95% CI: 0.42-0.99; *P* = 0.045) compared to those part of larger families (Table 3).

## Discussion

The current study was conducted in the South Omo Zone of southern Ethiopia to estimate the prevalence of LTBI among 497 pastoralists using house to house surveys. The prevalence of LTBI among the study participants was 50.5%. Being a resident of the Dasanech District and having a habit of eating raw meat were significantly associated with increased odds of being positive for LTBI. Being a member of a large family was significantly associated with reduced odds of being positive for LTBI compared to those with a family size below 5.

The prevalence of LTBI recorded in South Omo pastoral communities was higher than that documented by a previous study conducted on Addis Ababa University male students in Ethiopia [23] and lower than a study conducted on Afar pastoral communities in Ethiopia [12]. The prevalence was higher than those documented in surveys of village doctors in China [24], people living in border areas in Nuevo Leon and Tamaulipasin Mexico [25], individuals attending health care centers in Southern Taiwan [26], a population living in Danyang County, Jiangsu Province inChina [27] and immigrants in the USA [28]. Moreover, the estimated prevalence of LTBI in South Omo was higher than the estimated 23% worldwide prevalence of LTBI [29]. The other Ethiopian studies [12, 23] and the current one suggest that LTBI surveillance deserves more attention in the Ethiopian TB Control Program, particularly in the context of marginalized, pastoralist populations. We hypothesize that interventions minimizing the risk of progression of LTBI to active TB in marginalized pastoralist populations is of paramount importance for TB prevention and control in Ethiopia in large and in pastoral communities in particular.

One study previously reported a positive association between the prevalence of LTBI and age and suggested that cumulative exposure to MTBC increases the likelihood of future infection [30]. For the South Omo cohort, we did not observe an association of LTBI prevalence with a specific age group, as also stated in other publications [12, 16, 31]. The socio-demographic characteristics of participants in our study suggest that MTBC strains are ubiquitous in endemic areas like the South Omo Zone. All inhabitants ranging from young to middle-aged adults are susceptible to infection. Consistent with our data, a study conducted by Legesse et al. on an Afar pastoral community described the lack of an effect of gender, age and socio-demographic traits on detection of LTBI [12]. We hypothesize that hyperendemicity, co-morbidities and poor treatment-seeking behaviors of pastoral communities increase the risk of infection with MTBC strains. The reason for an especially high prevalence of LTBI in the Dasanech District may be a low TB detection rate (14.28%) compared to that in the Benatsemay District (62%) [21]. People who live in high TB burden settings are more likely to be infected with MTBC pathogens than those who live in low disease prevalence areas [32].

The association of LTBI with raw meat consumption that we observed may reflect zoonotic transmission but needs to be verified. In contrast to reports that being a household contact of a TB patient increases the risk of LTBI [21, 33, 34], the prevalence of LTBI among household contacts was not observed for pastoralists in South Omo (this study) and Afar [12]. LTBI prevalence associated with household contacts was 51.4% as reviewed from data pertaining to 41 studies by Padmanesan*et al.* [35]. Socio-demographic factors and recruitment methods (recruitment house-to-house versus patients in a health clinic) may significantly influence outcomes of LTBI diagnostic surveys. The choice of the LTBI diagnostic assay may also influence the results. An inverse relationship between the prevalence of LTBI and size of family was reported in an Indian study [36], in agreement with the results of our study. Similar data were reported in studies conducted in Botswana [37] and New York City [38]. We hypothesize that intimacy of contact rather than family size enhances the risk of disease transmission.

This study used an IGRA recommended for the screening of LTBI. Our data likely reflect the true LTBI prevalence in the entire Zone. Compared to the skin test, IGRA use is more convincing as the latter specifically detects *Mtb* infection. Our study’s unique feature was the cohort, a population of remote, ethnically diverse, pastoralists affected by poor infrastructure and public health services. It was limited by the fact that the subjects were not tested for HIV (we did not obtain ethical approval for HIV testing).

## Conclusions

The prevalence of LTBI recorded by the present study in South Omo pastoral communities was greater than that reported for many other regions in the world. It implies that the inhabitants are at a high risk of developing TB, justifying more extensive LTBI screening and potentially preventative treatment for the affected individuals.

## Abbreviations

χ^2^: Chi square
IFN-γ: Interferon gamma
AOR: Adjusted odds ratio
BCG: Bacille Calmette-Guerin
CI: Confidence interval
DOTS: Directly observed treatment short-course
ELISA: Enzyme-linked immunosorbent assay
HIV/ADIS: Human immunodeficiency virus infection and acquired immune deficiency syndrome
IGRA: Interferon gamma release assay
IU/ml: International unite per milliliter
LTBI: Latent tuberculosis infection
MDR: Multi-drug resistance
Mtb: *Mycobaterium tuberculosis*
MTBC: *Mycobacterium tuberculosis* complex
QFT-IGIT: QuantiFERON-TB Gold in-tube test
SPSS: Statistical package for the social sciences
U.S.: United States
vs: Versus
WHO: World health organization
